# Haemolytic uremic syndrome following fire ant bites

**DOI:** 10.1186/1471-2369-15-5

**Published:** 2014-01-08

**Authors:** Yi-Che Lee, Jyh-Seng Wang, Jeng-Chuan Shiang, Ming-Kai Tsai, Kai-Tai Deng, Min-Yu Chang, Hsi-Hao Wang, Li-Chun Ho, Yi-Ting Chen, Shih-Yuan Hung

**Affiliations:** 1 Division of Nephrology, Department of Internal Medicine, E-Da Hospital/ I-Shou University, No.1, Yida Rd., Yanchao Dist., Kaohsiung City 824, Taiwan; 2 Institute of Clinical Medicine, National Cheng Kung University Medical, Tainan, Taiwan; 3 Department of Pathology, Kaohsiung Veterans General Hospital, Kaohsiung, Taiwan; 4 Division of Nephrology, Department of Internal Medicine, Kaohsiung Armed Forces General Hospital, Kaohsiung, Taiwan

**Keywords:** Fire ant, Haemolytic-uremic syndrome, Plasma exchange, Renal failure, Venomous insects

## Abstract

**Background:**

Haemolytic-uremic syndrome (HUS) is a severe, life-threatening disease with symptoms such as haemolytic anaemia, renal failure, and a low platelet count. Possible aetiology includes bacterial infections, medication, post-hematopoietic cell transplantation, pregnancy, autoimmune disease, and acquired immunodeficiency syndrome.

**Case presentation:**

We report the case of a 21-year-old healthy man who developed acute renal failure caused by HUS. Typical symptoms of HUS combined with severe uraemia developed following a large local reaction after suspected *Solenopsis invicta* (fire ant) bites. He was successfully treated with plasma exchange and achieved complete recovery of renal function.

**Conclusion:**

This is the first case illustrating a serious systemic reaction of HUS to fire ant bites, and highlights this severe complication in patients who sustain fire ant bites.

## Background

Haemolytic-uremic syndrome (HUS) is a disease commonly preceded by *E. coli* O157:H7 or other Shiga toxin-producing bacterial infections, medication (chemotherapy agents, cyclosporine, tacrolimus, quinine, ticlopidine, clopidogrel, oral contraceptives, valacyclovir, etc.), post-haematopoietic cell transplantation, pregnancy, autoimmune diseases (antiphospholipid antibody syndrome, systemic lupus erythematous, scleroderma renal crisis), acquired immunodeficiency syndrome, and idiopathic form of HUS [1].

To the best of our knowledge, there is currently no report establishing the relationship between HUS and red fire ant bites. Koya et al. reported a case of rhabdomyolysis and acute renal failure after fire ant bites, but no presentation of HUS was noted [2]. In our case, a 21-year-old man developed acute renal failure and HUS following a large local reaction on the left arm resulting from fire ant bites. This unusual case demonstrates a close relationship between HUS and fire ant bites. We discuss the mechanisms of HUS caused by fire ant venom in detail.

## Case presentation

A 21-year-old healthy male solider was stationed at a seed troop with the task of eradicating fire ants for 1 year. He was bitten by ants that were highly suspected to be fire ants, 2 weeks before hospital admission. Immediate flare and wheal developed over the left arm, followed by erythematous, oedematous, and indurated reactions. A spiking fever occurred in 2 days. He received medical attention at a local clinic, and the local reaction subsided gradually. However, 5 days later, he developed severe nausea and vomiting. His urine output also gradually decreased. He visited a local hospital for treatment, where he was administered intravenous fluids and medication including acetaminophen and metoclopramide, but no improvement was noted. Thereafter, the patient was transferred to our hospital.

On presentation, physical examination revealed a pulse rate of 75 beats/min, blood pressure of 150/84 mmHg, body temperature of 37.0°C, and respiratory rate of 20 breaths/min. The pertinent positive findings included mild obesity (body mass index, 28.2 kg/m^2^) and pale conjunctivae. No oedema, icteric sclera, abdominal mass, or palpably enlarged kidney was noted.

**Figure 1:**
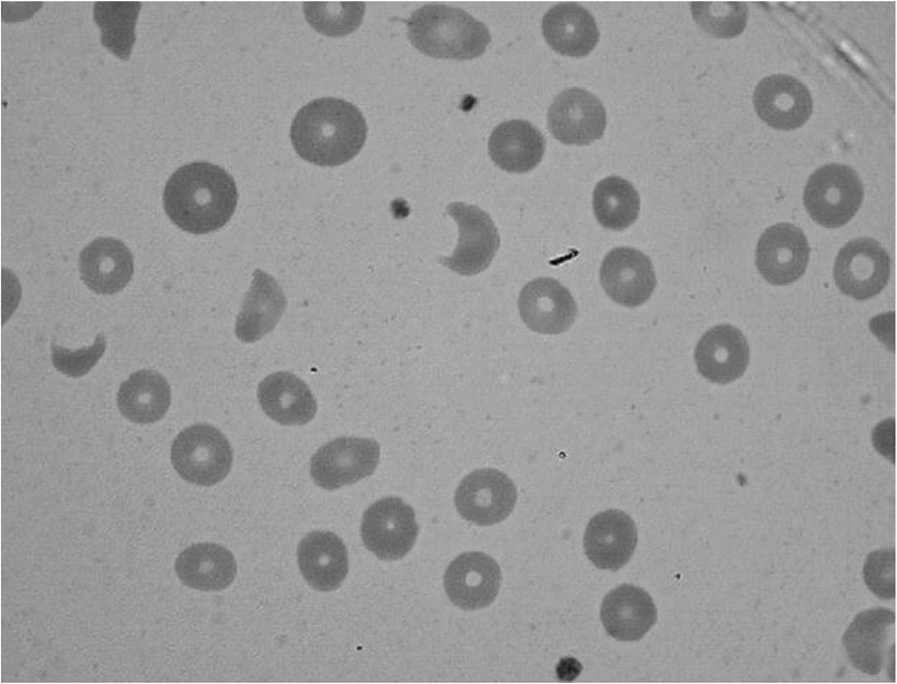
Peripheral blood smear showing some schistocytes.

Laboratory data on admission showed the following values: serum sodium, 128 mEq/L; potassium, 3.9 mEq/L; urea nitrogen, 153 mg/dL; creatinine, 20.1 mg/dL; calcium, 8.4 mg/dL; phosphorus, 3.9 mg/dL; albumin, 4.2 g/dL; aspartate aminotransferase, 127 U/L; alanine aminotransferase, 36 U/L; total bilirubin, 2.0 g/dL; direct bilirubin, 0.5 g/dL; uric acid, 19.1 g/dL; myoglobin, 422 ng/mL; lactate dehydrogenase, 3400 U/L; creatine kinase (CK), 503 U/L; CK-MB, 10.1 ng/mL; troponin, 0.01 μg/L; and haptoglobin <7.0 mg/dL. Arterial blood gases revealed a pH of 7.27, pCO2 of 41 mmHg, pO2 of 91 mmHg, oxygen saturation of 97.4%, and bicarbonate level of 18 mEq/L. Complete blood count (CBC) showed the levels of haemoglobin at 7.3 g/dL, white blood cells (WBC) at 8.8 × 10^3^/μL, platelets at 57 x 10^3^/μL, and a high reticulocyte level at 6.8%. WBC differential count showed neutrophils at 79.5%, eosinophils at 2.7%, basophils at 0.4%, and monocytes at 6.8%. Both prothrombin time and partial thromboplastin time were within normal ranges. Direct and indirect Coombs tests were both negative. A peripheral blood smear revealed 2–3 schistocytes per high-power field (Figure 1). Immunoglobulin (Ig) G, IgA, and IgM levels were all within normal ranges, but IgE level was high at 408.0 IU/mL. Serum complement showed the C3 level at 102 mg/dL (normal range, 90–180 mg/dL), and the C4 level at 22.6 mg/dL (normal range, 10–40 mg/dL). The serum antinuclear antibody level was within the normal range. Results for venereal disease research laboratory/rapid plasma reagin test, hepatitis B surface antigen (HBsAg), hepatitis C, and human immunodeficiency virus antibodies were all negative. Urinalysis revealed dark brown urine with 3+ protein, 0–3 red blood cells, and 3–5 white blood cells per high-power field, with positive results for haemoglobin. Renal ultrasound showed increased echogenicity (grade I) in both kidneys with right and left renal size at 12.4 × 5.45 cm and 12.5 × 5.4 cm, respectively.

**Figure 2:**
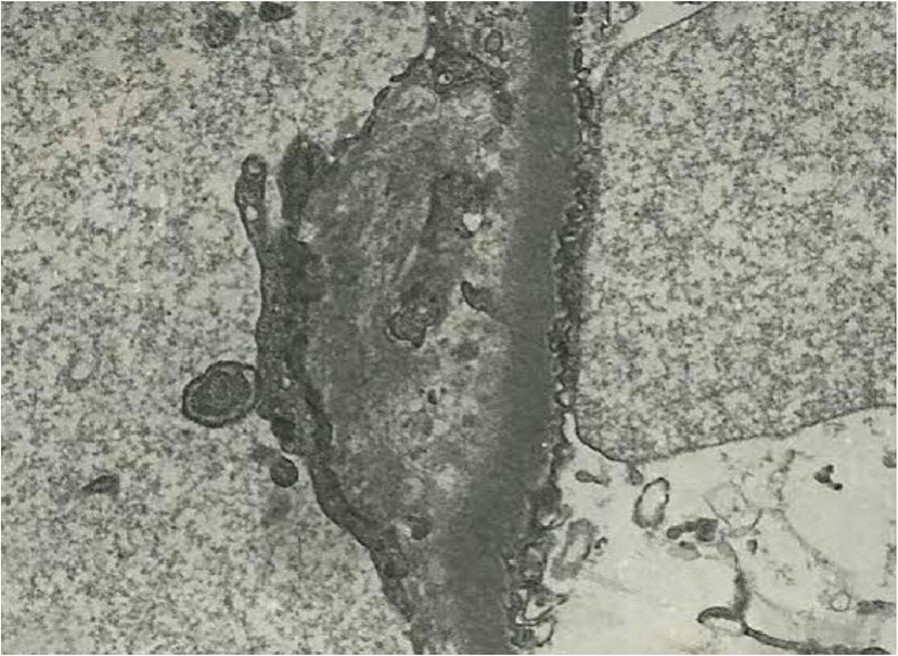
Electron microscopy of renal biopsy shows subendothelial widening with fluffy material and double contour formation of the glomerular basement membrane.

**Figure 3:**
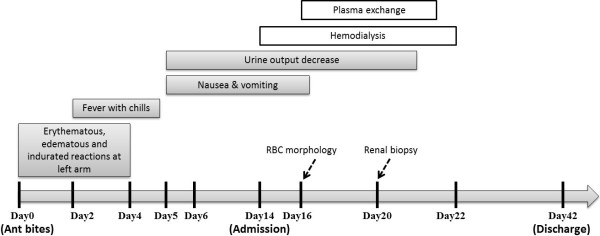
Timeline of the development of haemolytic-uremic syndrome following fire ant bites.

The patient was diagnosed with acute renal failure secondary to HUS and treated with plasma exchange with one plasma volume per day for a total of 6 days. Haemodialysis was also initiated for oliguria and uremic symptoms, including nausea, vomiting, and dyspnoea. The patient received haemodialysis therapy every second day for a total of 6 sessions. Blood cultures for bacteria and stool culture for *E. coli* O157 were negative. An echo-guided renal biopsy was performed on day 7 of admission. Pathological examination showed a partially collapsed glomerulus, endothelial cell swelling, subendothelial widening with fluffy material, and double-contour formation of the glomerular basement membrane, compatible with HUS (Figure 2). After plasma exchange, haemodialysis, and supportive treatment, the patient's urine output and renal function continued to improve. At the same time, haemolytic anaemia and thrombocytopenia also gradually improved and dialysis was terminated on the day 8 of admission. Finally, the patient was discharged with a creatinine level of 1.9 mg/dL (Figure 3).

On follow-up examination 1 month after discharge, the patient was in good health with normal renal function and CBC. Because of persistent hypertension (blood pressure around 140/90 mmHg), he was still on antihypertensive drugs (amlodipine 5 mg OD, carvedilol 25 mg OD) 1 year after discharge.

## Discussion

Historically, the red fire ant *S. invicta* was imported from South America into North America, residing in the soil of potted plants that were shipped in the 1930s. Since approximately the year 2000, the imported fire ant (IFA) has also been found in China and Taiwan. It belongs to the insect order *Hymenoptera*, which includes *Formicidae* (ants), *Vespidae* (wasps, hornets, and yellow jackets), and *Apidae* (honey bees and bumble bees) [3,4]. The venom composition of the IFA is complex, including several proteins capable of inducing allergy and anaphylaxis. In addition to these proteins, piperidine alkaloids are also present and are a major component of IFA venom (approximately 95%) [5]. This component is not allergenic but is responsible for the local pain and pustules commonly experienced in ant bites. After fire ant bites, three different reactions may be elicited: local, large local, or systemic (anaphylaxis) [6-9]. A local reaction results in an immediate flare and wheal followed by a pustule after approximately 1 day. A large local reaction is an erythematous, oedematous, and indurated region extending beyond the pustule, which may last several days. The occurrence rate of serious systemic reactions is much rarer, at approximately 2%.

The above complications relate to the biological activity of the ant venom, which causes cytotoxic, neurotoxic, and haemolytic reactions within the body [10,11]. It also activates coagulation and predisposes the individual to a hypercoagulable state [12-14]. Javors et al. discovered that fire ant venom alkaloids affect certain physiological and biochemical functions of human platelets and neutrophils in 1993 [14]. He found that venom alkaloids induced an increase in platelet intracellular calcium ion concentration (Ca^2+^), secretion of dense granules, and aggregation. The aggregation response was less complete than that of the platelet-activating factor (PAF). Furthermore, pre-treatment of platelets with venom alkaloids produced enhanced PAF-related intracellular Ca^2+^ spiking, suggesting synergism between the two agonists. Venom alkaloids also induce an increase and accumulation of neutrophils and intracellular Ca^2+^. These results suggest that fire ant venom alkaloids do activate platelets and neutrophils. Therefore, platelet thrombi formation and endothelial injury may occur *in vivo* after fire ant bites. In fact, there is a report on a 5-day-old neonate developing microangiopathic haemolytic anaemia after fire ant bites [15]. These findings may partially explain the pathogenesis of HUS following exposure to fire ant venom. There are some case reports of HUS or ADAMTS-13 deficiency after scorpion sting [16-19]; however, to the best of our knowledge, there are no existing reports of HUS development as a result of ant bites.

The limitation of our report is that we cannot examine the complement regulators, genetic mutations, or ADAMTS 13 activity in this patient. Therefore, the possibility of atypical HUS and TTP cannot be completely excluded. The diagnosis of atypical HUS is substantially one of exclusion, based on evidences of microangiopathic hemolytic anemia (MAHA), thrombocytopenia, and renal failure, in the absence of infections by Shiga-toxin producing bacteria or other micro-organisms associated with HUS, of possible causes of secondary forms of HUS (such as medications, autoimmune diseases, and malignancy) and of reduced ADAMTS 13 activity (< 10%) [20]. Atypical HUS designates a primary disease due to a disorder in complement alternative pathway regulation. The onset is from the neonatal period to adult [20,21]. At the first episode of HUS, about one-third of patients have progressed to end-stage renal disease and half of the patients have relapses. Gene mutations—including complement regulatory proteins thrombomodulin, factor H, factor I, and membrane cofactor protein (MCP)—have been demonstrated. Mutations in the genes for C3 convertase proteins, C3 and factor B, as well as patients with anti-factor H antibodies, have also been reported [20,21]. The disease is familial in approximately 20% of pedigrees. Recent studies have showed that the complement abnormalities, such as reduced C3 levels, reflecting activation of complement alternative pathway, are found only in a subset of patients, and are not necessary to make the diagnosis of atypical HUS [20,22]. Although our patient didn’t have HUS-like episodes family history and had normal serum C3, C4 levels, we still cannot rule out a putative role of complement dysregulation in the pathogenesis of this case. ADAMTS 13 activity is typically decreased or absent in thrombotic thrombocytopenic purpura (TTP) patients, and is a good marker for differential diagnosis between TTP and atypical HUS. There has been a case report of ADAMTS 13 deficiency after a scorpion sting and successful recovery after treatment by plasma exchange [19]. And, a consistent subset of patients with severe ADAMTS13 deficiency (e.g. 29% in the series of 65 patients from the Oklahoma TTP-HUS Registry presented by JN George) has no neurological symptoms [23]. The clinical features of TTP are similar to those of our case, so we think although there were no neurologic symptoms (including headache, dysphasia, seizure, confusion, stupor, or coma) in our patient; TTP remains a possible differential diagnosis unless normal ADAMTS13 activity is proved.

## Conclusions

We present the first case report of HUS and acute renal failure resulting from fire ant bites. The venom alkaloids of the ants may be responsible for the activation of platelets and neutrophils, the formation of platelet thrombi, endothelial injury, and ultimately the development of HUS. Although the possibility of atypical HSU and TTP cannot be completely excluded, this case still illustrates a serious systemic reaction of HUS to fire ant bites and highlights the need for awareness of this complication in patients who sustain fire ant bites.

## Consent

Written informed consent was obtained from the patient for publication of this case report and any accompanying images.

## Abbreviations

CBC: Complete blood count; CK: Creatine kinase; HUS: Hemolytic-uremic syndrome; HBsAg: Hepatitis B surface antigen; Ig: Immunoglobulin; IFA: Imported fire ant; PAF: Platelet-activating factor; TTP: Thrombotic thrombocytopenic purpura.

## Competing interests

The authors declare that they have no competing interests.

## Authors’ contributions

All the authors were involved in the clinical management of the case and the writing of the report. All authors read and approved the final manuscript.

## Pre-publication history

The pre-publication history for this paper can be accessed here:

http://www.biomedcentral.com/1471-2369/15/5/prepub
